# The Integrated Nutrition Pathway for Acute Care (INPAC): Building consensus with a modified Delphi

**DOI:** 10.1186/s12937-015-0051-y

**Published:** 2015-06-19

**Authors:** Heather H Keller, James McCullough, Bridget Davidson, Elisabeth Vesnaver, Manon Laporte, Leah Gramlich, Johane Allard, Paule Bernier, Donald Duerksen, Khursheed Jeejeebhoy

**Affiliations:** 1 Schlegel- University of Waterloo Research Institute for Aging, University of Waterloo, Waterloo, Canada; 2 Department of Kinesiology, University of Waterloo, Waterloo, Canada; 3 Canadian Malnutrition Task Force, Canadian Nutrition Society, Ottawa, Canada; 4 Department of Family Relations and Applied Nutrition, University of Guelph, Guelph, Canada; 5 Réseau de Santé Vitalité Health Network, Campbellton, NB Canada; 6 Department of Medicine, University of Alberta, Alberta Health Services, Edmonton, Canada; 7 Department of Medicine, University Hospital Network,University of Toronto, Toronto, Canada; 8 Jewish General Hospital, Montréal, Canada; 9 Department of Medicine St-Boniface Hospital, University of Manitoba, Winnipeg, Canada; 10 Department of Medicine St-Michael’s Hospital, University of Toronto, Toronto, Canada

**Keywords:** Malnutrition, Nutrition screening, SGA, Acute care, Evidence, Care pathway, Delphi survey

## Abstract

**Background:**

Malnutrition is commonly underdiagnosed and undertreated in acute care patients. Implementation of current pathways of care is limited, potentially as a result of the perception that they are not feasible with current resources. There is a need for a pathway based on expert consensus, best practice and evidence that addresses this crisis in acute care, while still being feasible for implementation.

**Methods:**

A modified Delphi was used to develop consensus on a new pathway. Extant literature and other resources were reviewed to develop an evidence-informed background document and draft pathway, which were considered at a stakeholder meeting of 24 experts. Two rounds of an on-line Delphi survey were completed (n = 28 and 26 participants respectively). Diverse clinicians from four hospitals participated in focus groups to face validate the draft pathway and a final stakeholder meeting confirmed format changes to make the pathway conceptually clear and easy to follow for end-users. Experts involved in this process were researchers and clinicians from dietetics, medicine and nursing, including management and frontline personnel.

**Results:**

80 % of stakeholders who were invited, participated in the first Delphi survey. The two rounds of the Delphi resulted in consensus for all but two minor components of the Integrated Nutrition Pathway for Acute Care (INPAC). The format of the INPAC was revised based on the input of focus group participants, stakeholders and investigators.

**Conclusions:**

This evidence-informed, consensus based pathway for nutrition care has greater depth and breadth than prior guidelines that were commonly based on systematic reviews. As extant evidence for many best practices is absent, the modified Delphi process has allowed for consensus to be developed based on better practices. Attention to feasibility during development has created a pathway that has greater implementation potential. External validation specifically with practitioner groups promoted a conceptually easy to use format. Test site implementation and evaluation is needed to identify resource requirements and demonstrate process and patient reported outcomes resulting from embedding INPAC into clinical practice.

## Background

Malnutrition is a well-known problem in acute care hospital patients; it is estimated that the prevalence at admission is between 30-45 %, with older adults more likely to be malnourished [1–3]. Malnutrition results in longer length of stay and other negative health outcomes [3–5]. Staying longer in hospital may also perpetuate malnutrition due to dissatisfaction with food [6], exposure to infectious agents [7], and decreased mobility and function [8]. Despite the recognition of the importance of nutrition to the recovery of patients, relatively little intervention research, outside of nutritional supplementation, has been conducted [9].

Prior work suggests that individualized care and monitoring can improve food intake and health outcomes [9]. For example, Feldblum et al. [10] demonstrated that in-hospital and home dietitian visits, prescriptive diets and use of oral nutritional supplements (ONS), improved nutritional status as measured with the Mini Nutritional Assessment. Care that starts in the hospital and follows through with transitions to the community has also been shown to improve functional outcomes [11]. Thus, quality nutrition care that promotes individualized approaches for malnourished patients is a best practice [12, 13] that has benefits to the patient and potentially the health system. The challenge is in identifying those patients who would benefit from this individualized care.

Recent Canadian research suggests that the traditional reasons for referral to the specialized and limited resource of the dietitian are not standardized. Ad-hoc methods are typically used to identify patients whom the dietitian should consult [14]. During the first few days of admission, diagnoses and diet orders drive consultations, whereas complications including constipation and dysphagia resulted in a referral to the dietitian four or more days after admission. Malnutrition, even severe, was a poor predictor of consulting a dietitian, confirming that physicians and nurses need a structured screening tool and process to support their identification of nutrition risk [14]. Integration of simple, valid and reliable screening tools into clinical practice is essential to identify malnourished patients [15].

To promote best practice, guidelines have been developed that recommend nutrition screening on admission followed with a comprehensive assessment for those at risk [16–19]. The European Society for Parenteral and Enteral Nutrition (ESPEN) guideline emphasizes screening and choice of screening tools; assessment, monitoring, and communication, including transitions out of hospital, are also discussed [18]. Although this guideline suggests that not all ‘at-risk’ patients receive assessment and individualized treatment, little direct guidance is provided for making the determination of who needs this individualized approach [18]. More recently, an algorithm described in a review article by the feedM.E. International group suggests that treatment in the form of diet counselling, food fortification or oral nutritional supplements should occur for all ‘at risk’ patients whose disease condition may result in further malnutrition, regardless of confirmation of malnutrition [19]. This early treatment strategy is consistent with the ESPEN guideline that states a ‘nutrition plan’ should be developed post-identification of risk [18]. Yet, as nutrition screening tools do not diagnose malnutrition and typically have higher sensitivity than specificity [20], such a strategy could have significant resource and cost implications due to a high level of false positives. In contrast, the American Society for Parenteral and Enteral Nutrition (A.S.P.E.N.) guideline states that screening should always be followed by a comprehensive assessment to diagnose malnutrition and determine if interventions are required [16]. The ESPEN and A.S.P.E.N. guidelines describe this comprehensive assessment, emphasizing its level of detail, variety of indicators and diverse information required (diet, anthropometry/body composition, biochemistry, function, clinical and social information) to determine nutritional status and treatment approaches [16, 18].

A noted challenge with screening and requiring a comprehensive individualized assessment to diagnose and identify a treatment plan for malnourished patients is that current skilled nutrition professionals (i.e., dietitians) who can conduct these assessments are a limited resource [14, 15, 21] and screening will overload this service. As upwards of one in two patients admitted to medical and surgical wards in hospital could screen positive [3], this is a valid concern. Yet, it is noted that malnutrition will continue to be underdiagnosed and undertreated without valid and practicable pathways implemented routinely in hospitals [22, 23]. A feasible and sustainable algorithm that is evidence based is needed. Additionally, although a variety of practice recommendations suggest the need for an interdisciplinary approach where all staff is ‘food aware’ and where preventative practices are put into place to support food intake [15, 24–27], the current guidelines [16–19] do not emphasize strategies that support a team approach to addressing malnutrition.

Based on the recent work of the Canadian Malnutrition Task Force (CMTF) and the Nutrition Care in Canadian Hospitals study, the following study was conducted with the primary research objective of creating an evidence-informed consensus-based pathway for nutrition care that is feasible and sustainable and promotes roles and accountability across the interdisciplinary team. Focus was placed on medical and surgical units and older adult patients. The ultimate aim of this work is to provide acute care practitioners with a step-by-step nutrition care program based on this pathway that can enhance the care and improve clinical outcomes of patients through evidence based practice.

## Methods

The Delphi method is a process designed to achieve consensus in which expert opinion is gathered in a systematic way through multiple rounds of surveys [28–30]. This approach provides for simultaneous, anonymous input from experts and is advantageous when participants are geographically dispersed [28]. Typically, three or four rounds are used [28, 30]. The first allows the experts to define the questions/issues, and then subsequent rounds allow them to work through a series of statements or questions using a standardized format to develop consensus [30]. Reporting back results from each round to the experts is a key component towards building consensus, allowing participants to evaluate their answers in light of ratings from the rest of the group [28, 30].

**Fig. 1:**
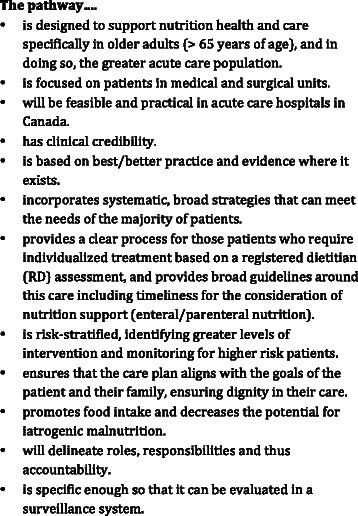
Guiding Principles for Development of the Integrated Nutrition Pathway for Acute Care (INPAC)

**Fig. 2:**
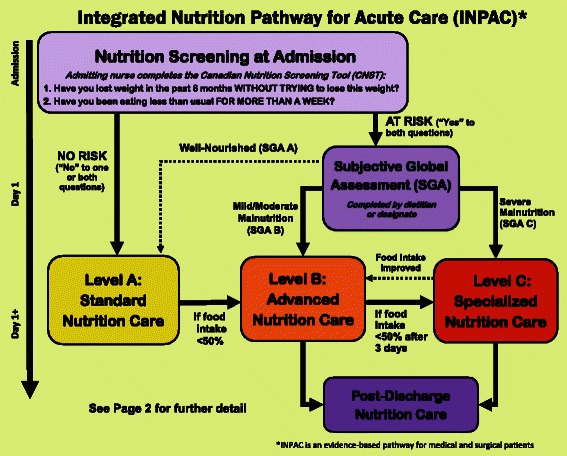
Overview of the Integrated Nutrition Pathway for Acute Care (Page 1)

A modified Delphi process was used to achieve the objective of this study. An initial face-to-face meeting aligned experts with the purpose of the consensus building process; this was followed by two anonymous survey rounds using an internet platform. As other algorithms or guidelines were available as a starting point, with some based on graded systematic reviews of the literature [16, 17], initial evidence synthesizing efforts were only undertaken to extend and update this literature. The following steps were completed in this modified Delphi process: 1) the investigators agreed upon a set of principles to guide the development of the algorithm (Fig. 1); 2) a small working group of investigators scanned the extant literature to identify key resources on better nutrition care practices; 3) websites of key professional organizations were scanned and pertinent documents and resources were reviewed, 4) a draft pathway was created based on these reviews and inputs; 5) an expert stakeholder meeting was held to discuss and confirm the issues and the purpose of the consensus building process; agreement for principles behind development of the pathway was attained and the draft was reviewed; 6) the pathway was revised; 7) two rounds of an on-line survey were completed to revise and reach consensus on the pathway; 8) clinical staff from a medical or surgical unit in four hospitals participated in focus groups to face validate the pathway, and 9) a final stakeholder meeting confirmed the final version of the Integrated Nutrition Pathway for Acute Care (INPAC).

### Identification of key literature and development of draft pathway

Two investigators (HK, BD) conducted an evidence and best-practice scan of the literature, using numerous sources including peer-reviewed literature, as well as websites of leading organizations with a mandate consistent with ameliorating malnutrition in the acute care setting (e.g. American Society of Parenteral and Enteral Nutrition, Academy of Nutrition and Dietetics, European Society of Parenteral and Enteral Nutrition, National Institute for Health and Care Excellence, British Association for Parenteral and Enteral Nutrition, Fight Malnutrition Now, etc.). Search terms included: ‘nutrition care’, ‘acute care’, ‘malnutrition’, ‘screening’, ‘assessment’, ‘treatment plans’ and their derivations. The search was focused on citations after 2011 when the A.S.P.E.N. guideline, which was based on a systematic review of the literature, was produced [16]. Google Scholar and PubMed were the primary search engines used. All authors provided input into identifying key sites, review papers, guidance documents, position papers and other forms of pertinent evidence for consideration. Key journal (e.g. Clinical Nutrition, Journal of Human Nutrition and Dietetics, Journal of Parenteral and Enteral Nutrition) archives from 2010 onwards were also searched for pertinent titles. Evidence was accumulated into a background document and a draft pathway. Additionally, the experience of the CMTF in its own research on nutrition care processes was translated into the draft [3, 14, 15, 31–33]. This initial pathway and background document were vetted by a small working group of investigators (PB, ML, JM, LG) for refinement prior to the expert stakeholder meeting.

### Initial stakeholder meeting

Canadian expert stakeholders were carefully selected to ensure: disciplinary representation; roles in the acute care setting (e.g. clinician, manager); research expertise in the content area; and regional representation. There were 24 experts and four facilitators in attendance (Table 1). The moderator was the lead investigator who has experience in consensus building processes [34, 35] as well as expertise in the subject matter; this combination of expertise is recommended for Delphi methods [30]. Each expert was sent the background document, draft algorithm, agenda for the meeting, and attendees for the day. The background document was essential, as it ensured that all participants in the meeting would have a common base of knowledge with respect to the issues of nutrition care in hospitals, avoiding a common challenge of experts relying on their own reading and experience with the literature to inform their decisions [28]. The draft pathway was briefly presented and agreement was attained with the principles required to develop the algorithm (Fig. 1). Using a poster version of the pathway, experts were asked to independently identify points that they believed were most contentious and should be the focus of discussion for the rest of the day. They were each provided with four colour coded dots that determined their priority for discussion, to affix to the poster board depicting the draft pathway. As a result of this voting, the key areas in priority order for discussion were: the standard nutritional treatment provided to prevent iatrogenic malnutrition; nutritional risk screening upon admission and subsequent actions; monitoring of patients with “no nutrition risk”; and post discharge care practises. Facilitators led the discussion at three tables that had been pre-set to ensure regional and professional diversity; this discussion took approximately 3 h. After timed intervals of discussion, facilitators reported key novel points in a round-robin fashion, identifying how the draft algorithm could be modified for these specific areas or other considerations. The day was completed with a summary of the modified Delphi process to occur over the next three months.

**Table 1:** Characteristics of Stakeholder Meeting Participants and Focus Groups

Characteristic	% (n)		
	Initial stakeholder	Final stakeholder	Focus groups
	(*n* = 24)	(*n* = 25)	(*n* = 47)
Professions			
Physician	29.2 (7)	20 (5)	4.3 (2)
Dietitian	54.2 (13)	60 (15)	27.7 (13)
Nurse	12.5 (3)	8 (2)	38.3 (18)
Other	4.2 (1)	12 (3)	29.9 (14)
Current Role*			
Clinician	75 (18)	56 (14)	100 (47)
Management	25 (6)	28 (7)	
Researcher	37.5 (9)	20 (5)	
Advocate/stakeholder	16.7 (4)	12 (3)	
Region			
Quebec + east provinces	12.5 (3)	20 (5)	21.3 (10)
Ontario + Manitoba	66.7 (16)	56 (14)	36.2 (17)
Western provinces	20.8 (5)	24 (6)	42.5 (20)

*note some individuals held more than one role, thus % > 100 %

Immediately upon completion of the face-to-face expert meeting, notes from facilitated discussions were summarized and the pathway revised by a small working group (HK, BD, JM, EV). The points of discussion around contentious issues became the basis for the first on-line Delphi survey questions; additionally questions were asked on each step of the pathway to confirm consensus noted during the stakeholder meeting (e.g. “All screened patients should have malnutrition confirmed.”). Additionally it was identified at the expert meeting that greater diversity in representation was needed for subsequent steps in building consensus, especially from front-line nurses. Using the networks of the stakeholders to make these connections, additional individuals interested in the process were also invited to take part in the next phase using on-line surveys.

### On-line surveys to build consensus

Two rounds of online surveys were used to ask the expert stakeholders specific questions about every individual step in the pathway. Questions were developed by three of the investigators who did not complete the survey (HK, BD, JM). It was decided a priori by the investigators that consensus would be defined as any question/issue in which at least 80 % agreement (totally or somewhat agree) was reached [30, 36]. Thirty-five experts and stakeholders were invited by email, with a total of 28 participants for round one (80 %) (Table 2) and 26 for round two. Co-investigator participants (JA, PB, ML, LG, KJ, DD) completed the on-line surveys as they are leading experts in Canada. As all participants account for only one vote or rating for questions, their expert status did not carry any undue weight in the total of 26 participants. Furthermore, qualitative comments were treated equally and fed back to participants anonymously; thus the greater expertise of the investigators did not unduly influence the consensus process. These qualitative comments were also used to revise the pathway and develop questions for the next survey. A single gift certificate incentive ($100) was provided for participants. If a participant completed both rounds of the survey, their name was included in a list from which a random selection was made using their participant number.

**Table 2:** Demographics of Delphi Survey Participants, Round 1 (*n* = 28)

Gender	Female		Male		No Response
	75 % (*n* = 21)		14 % (*n* = 4)		11 % (*n* = 3)
Age	25-34	35-44	45-54	55-64	65+
	7.1 % (2)	14.3 % (4)	42.9 % (12)	28.6 % (8)	7.1 % (2)
Discipline	Dietitian		Physician	Nurse	Other
	60.7 % (17)		25.0 % (7)	10.7 % (3)	3.6 % (1)
Current Position	Direct Care in Acute Care Hospital		Other		No Response
	46.4 % (13)		50.0 % (14)		3.6 % (1)
Years in Current Position	< 5 yrs		5-9 yrs	10-19 yrs	20+ yrs
	21.4 % (6)		17.9 % (5)	32.1 % (9)	28.6 % (8)

An invitation email was sent to all potential participants with a link to a FluidSurveys platform (www.fluidsurveys.com). Instructions, decision rules on how consensus would be achieved and timelines for completion of the survey were provided on the introductory page. Participants were provided just over three weeks to complete each round and an automated email reminder was sent twice to participants reminding them to submit their survey results after the second and third week. The introductory page also requested demographics for each participant (age group, gender, discipline, current position, and duration of time in that position). Each subsequent page of the survey included the draft pathway for easy reference, as well as questions categorized under each component of the algorithm (e.g. admission screening).

In the first Delphi survey round, 41 questions were posed and participants could revisit questions if desired before their submission. A four-point scale was used for rating of agreement with the item (totally agree to totally disagree); a neutral option was avoided to force participants to indicate their agreement or disagreement with an item. Some questions also included defined response options. For example, these questions were focused on specific details such as roles of different staff or timing of transition points in the pathway. After each question, the participant had the opportunity to provide comments on why they provided their ranking. An open-ended question was an option at the end of the survey for any further comments from participants.

After the first round of the survey, the draft pathway was edited based on where consensus was reached. Structured questions based on areas lacking consensus and the comments from round 1 helped to clarify the 23 questions posed in round two. The second round occurred approximately 3 weeks after the first round was completed. Two participants were allowed to delay their responses for the second round due to vacation time. FluidSurveys provided details on participants for each round (% with each characteristic), proportion with various agreement ratings and qualitative comments per question. The results of each question were exported to a text file for easy review, analysis and summarization by the analysis team (HK, JM). Qualitative content analysis [36] was used for comments, with minimal interpretation and these results were used to refine questions for round two; there were fewer qualitative comments from round two of the survey.

### Focus groups and final stakeholder meeting to finalize the INPAC

Once the pathway was defined with the modified Delphi process, four diverse hospitals (region, academic vs. community) were recruited to face validate the algorithm. Clinical staff on medical or surgical units in each hospital were invited to take part in a one-hour focus group (*n* = 5 groups *n* = 47 participants). Participants were from nursing, dietetics, allied health (e.g. speech language pathology) and food service. Questions and discussion were focused on: relevance of the pathway to improve nutrition care practice; any gaps in the pathway that need to be addressed; potential changes to the presentation of the pathway to make the interpretation easier for clinicians; and discussion on implementation aspects, such as resource utilization and role delegation. Participants were provided the pathway and its instructions before the focus group for their review. Group discussion was audiorecorded but not transcribed. Three investigators (HK, BD, EV) independently listened to each focus group (excepting the Francophone group which was only analyzed by EV, who is bilingual), making detailed notes on how the pathway required modification. They met to discuss these modifications, which were then included in a revision of the INPAC.

This revised INPAC was then presented to a stakeholder group, many of whom were participants at the initial meeting, to confirm these revisions and make any other formatting suggestions (e.g. final name). During this one-day face-to-face meeting, the project lead presented the results to date including the suggested modifications by the clinical focus group participants. Stakeholders from diverse disciplines were allocated to small tables for discussion of the revised pathway and main points on any further revisions were brought before the whole group; voting was taken to determine the final name of the algorithm. An ethics board at the University of Waterloo cleared this research (ORE# 19890) and the ethics boards at each hospital where a focus group was assembled also approved this aspect of the work.

## Results

Tables 1 and 2 present the demographics of stakeholder meeting and focus group participants and those who completed the Delphi survey at round one. Conduct of the Delphi over the summer months likely influenced participation rate, but 80 % of invitees responded, demonstrating a high level of engagement with the subject matter. It is not surprising that the majority of participants were dietitians as they have the greatest role in nutrition care in hospitals in Canada. Frontline providers made up 60 % of stakeholders at the face-to-face meeting, while a slightly higher proportion of management was included in the on-line survey. This is likely due to more invitations being extended to dietitians who worked in food service and nurses working in management based on recommendations from the first stakeholder meeting. Almost two-thirds (60 %) of Delphi respondents had 10+ years of experience in their current position indicating that these were experts in their own area or practice. Focus group participants were predominately nursing and nutrition staff, with other allied staff participating in each group; average years working was over 10. The final stakeholder meeting included 25 participants and four research staff with nursing, nutrition and medicine represented.

Approximately half of the draft pathway achieved consensus in the first round of the Delphi survey. Table 3 presents those areas that achieved consensus (at least 80 % somewhat/totally agree) in round one. Some notable points were: 1) some patients should bypass screening due to their need for nutrition care or incapacity to complete the screening (e.g. comatose, language barrier); 2) subjective global assessment (SGA) [37] is preferably completed by a dietitian; 3) food intake and body weight should be measured as part of nutrition monitoring for all patients and patient/family/health care aide can monitor food intake with a standardized form when applicable; and 4) nutrition discharge plans are preferably completed by the dietitian. Of note, some qualitative comments provided for these questions suggested that although there was consensus, some flexibility in the pathway was needed. For example, some experts were concerned about the automatic liberalization of diets for all lower priority malnourished patients. As a result, the final algorithm provides flexibility in the Advanced Nutrition Care strategies.

**Table 3:** Delphi Round One- Consensus Results

Question/Statement	% Totally Agree (N)	% Somewhat Agree (N)	% Somewhat Disagree (N)	% Totally Disagree (N)	% Totally/Somewhat Agreed
Nutritional screening is necessary upon admission for all non-traumatic medical and surgical patients.	82.1 (23)	17.9 (5)	0	0	100
Pre-admission screening is appropriate for elective admissions. (*n* = 27)	74.1 (20)	22.2 (6)	3.7 (1)	0	96.3
If deemed nutritionally “at risk” after initial screening, a subjective global assessment (SGA) will be completed.	71.4 (20)	21.4 (6)	7.1 (2)	0	92.9
If SGA classifies a patient as moderately malnourished ('B') but a lower priority for individualized assessment and treatment, Advanced Nutrition Care strategies should be implemented (i.e. higher protein diet).	67.9 (19)	28.6 (8)	3.6 (1)	0	96.4
If SGA classifies a patient as "severely malnourished" ('C'), the patient should be referred to the RD for comprehensive assessment and individualized treatment.	100 (28)	0	0	0	100
Nutrition care of patients referred for comprehensive assessment should be individualized based on the treatment plan prescribed by the RD.	82.1 (23)	17.9 (5)	0	0	100
Nutrition monitoring of patients referred for comprehensive assessment should be individualized based on the treatment plan prescribed by the RD.	78.6 (22)	14.3 (4)	3.6 (1)	3.6 (1)	92.9
Frequency of monitoring should increase with increased level of nutritional risk/malnutrition. (*n* = 27)	77.8 (21)	22.2 (6)	0	0	100
All non-traumatic medical/surgical patients should have their body weight measured at admission.	85.7 (24)	14.3 (4)	0	0	100
Body weight should be measured regularly as a gauge for changes in nutritional status in all non-traumatic medical/surgical patients.	60.7 (17)	21.4 (6)	17.9 (5)	0	82.1
For patients admitted as low-risk/well-nourished, artificial food & nutrition (AFN) should be considered if intake is suboptimal for 7-10 days post admission. (*n* = 27)	63.0 (17)	25.9 (7)	11.1 (3)	0	88.9
For patients admitted as malnourished, AFN should be considered if intake is suboptimal for 3 days post-admission. (*n* = 26)	53.8 (14)	30.8 (8)	15.4 (4)	0	84.6
If a patient was identified as malnourished (SGA B/C) on admission, the patient/family should be provided with recommendations to improve nutritional status post discharge.	89.3 (25)	7.1 (2)	3.6 (1)	0	96.4
If nutrition is still an issue at discharge, nutrition transfer recommendations should be embedded in discharge communications for their community health care professionals.	100 (28)	0	0	0	100

**Fig. 3:**
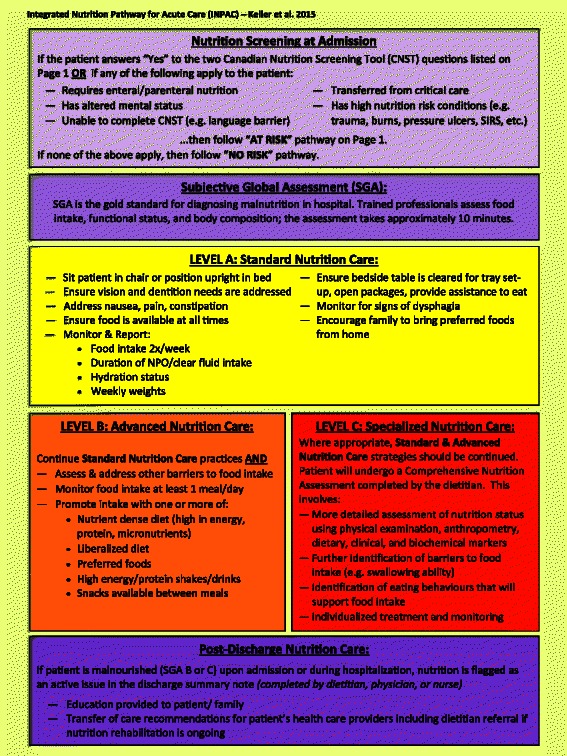
Detail on key components of the Integrated Nutrition Pathway for Acute Care (Page 2)

Table 4 shows the results of all items asked in round two. Of note, role delineation for various pathway activities was a key area for this round. For example, the diet technician was seen as an individual who could be responsible for review of food intake forms and determination of suboptimal intake. All of the 23 questions asked in round two achieved consensus except for two: NPO (no food by mouth) for 3 or more days results in an automatic referral to the dietitian for assessment, and monitoring of food intake daily for patients receiving Advanced Nutrition Care. These two areas, as well as qualitative comments from the participants in both rounds were discussed by the investigator team to determine how or if these issues would be considered in the final pathway. The final pathway is presented in Fig. 2 and Fig. 3 (note further guidance on using the algorithm is provided at www.nutritioncareincanada.ca).

**Table 4:** Delphi Round Two- Consensus Results (*n* = 26)

Question/Statement	% Totally Agree (N)	% Somewhat Agree (N)	% Somewhat Disagree (N)	% Totally Disagree (N)	% Totally/Somewhat Agreed
Where feasible, nutrition screening can be completed by the patient as part of the pre-admit documentation. (*n* = 25)	68.0 % (17)	24.0 % (6)	8.0 % (2)	0	92.0 % (23)
For non-elective admissions, nutrition screening occurs on day 1 or 2 of admission.	73.1 % (19)	11.5 % (3)	3.8 % (1)	11.5 % (3)	84.6 % (22)
SGA should be completed within 24 h of screening.	53.8 % (14)	38.5 % (10)	7.7 % (2)	0	92.3 % (24)
If patient is classified as SGA ‘C’, RD comprehensive assessment occurs on the same day.	46.2 % (12)	50.0 % (13)	3.8 % (1)	0	96.2 % (25)
Potential treatment options for the Advanced Nutrition Care should be flexible/individualized to the patient/setting.	76.9 % (20)	11.5 % (3)	3.8 % (1)	7.7 % (2)	88.5 % (23)
NPO status should be monitored on a daily basis.	80.8 % (21)	19.2 % (5)	0	0	100 % (26)
Being NPO for 3 days necessitates an RD comprehensive assessment (*n* = 25)	52.0 % (13)	24.0 % (6)	12.0 % (3)	12.0 % (3)	76.0 % (19)
In low-risk/well-nourished patients, food intake is monitored on Day 3 and 5 of admission using a meal intake form completed by the patient/family or Health Care Aid/Diet Technician.	46.1 % (12)	34.6 % (9)	19.2 % (5)	0	80.8 % (21)
If a low-risk patient has suboptimal food intake on day 3, they should be moved to Advanced Nutrition Care. (*n* = 24)	58.3 % (14)	33.3 % (8)	8.3 % (2)	0	91.2 % (22)
Suboptimal oral food intake for low-risk patients should be defined as <50 % of the meal. (*n* = 25)	56.0 % (14)	32.0 % (8)	8.0 % (2)	4.0 % (1)	88.0 % (22)
Patients receiving Advanced Nutrition Care should have their food intake monitored at minimum one meal per day.	46.2 % (12)	26.9 % (7)	19.2 % (5)	7.7 % (2)	73.1 % (19)
Lower-priority moderately malnourished (SGA 'B') patients receiving Advanced Nutrition Care should receive a comprehensive RD assessment if their food intake is suboptimal after two days of receiving standard treatment(s).	46.2 % (12)	42.3 % (11)	11.5 % (3)	0	88.5 % (23)
Suboptimal oral intake for lower priority B patients is defined as <50 % of the meal. (*n* = 25)	52.0 % (13)	40.0 % (10)	4.0 % (1)	4.0 % (1)	92.0 % (23)
Low-risk patients should have their body weight measured at minimum once/week.	65.4 % (17)	19.2 % (5)	15.4 % (4)	0	84.6 % (22)
Lower priority SGA B patients should have their body weight measured at minimum once/week.	65.4 % (17)	26.9 % (7)	7.7 % (2)	0	92.3 % (24)
Food intake, and not change in body weight, is the primary mechanism for determining a change in nutrition care (e.g. from Standard Nutrtion Care to Advanced Nutrition Care) for low risk and lower priority SGA B patients.	76.9 % (20)	19.2 % (5)	3.8 % (1)	0	96.2 % (25)
Suboptimal oral intake for consideration of AFN should be < 50 % of offered meals and supplements. (*n* = 25)	52.0 % (13)	44.0 % (11)	4.0 % (1)	0	96.0 % (24)

Focus groups with frontline clinicians and service providers confirmed the pathway. The format was a common discussion point as well as how to implement the various core components of the INPAC within an individual hospital or unit. No core component of the INPAC was seen as inappropriate or requiring modification to promote quality practice. All aspects were seen as feasible with adequate training and structuring of care processes considered unique to each hospital. The pathway was revised to be more streamlined and easier to visually follow with instructions condensed and focused. It was noted that specific disciplinary roles (e.g. nursing) may require their own specific algorithm to ensure that all steps are implemented in practice. The final stakeholder meeting further refined the format of the pathway to ensure understanding of key concepts. For example, labeling of levels was considered instrumental to support interpretation by clinical groups.

## Discussion

The developed Integrated Nutrition Pathway for Acute Care (INPAC) amalgamates the best of the evidence to date on how to provide quality nutrition care to medical and surgical patients. As compared to published guidelines, several distinctions can be made. The INPAC provides greater detail than the ESPEN Screening Guideline [18], on timing of aspects of the care process as well as details on monitoring and preventative treatment for all patients, and not only those identified to be malnourished. Additionally, using SGA to diagnose malnutrition, the INPAC provides a step in between risk identification and the detailed, time-consuming comprehensive assessment [37]. Consistent with feedM.E. [19], INPAC also advocates use of SGA for the diagnosis of malnutrition, but unlike feedM.E., INPAC follows this with a comprehensive nutrition assessment for those patients who are severely malnourished and require individualization of their treatment plan. Without a comprehensive assessment, determination of an appropriate treatment plan for these patients is challenging. FeedM.E. [19] only considers how much, what route and type of nutritional support formula should be used to treat the malnutrition diagnosed by SGA. Consistent with the A.S.P.E.N. guideline [16], the INPAC promotes monitoring of nutritional status to ensure that developing malnutrition is not overlooked and that discharge planning and continuity of care occur. Yet, as with the ESPEN guideline, timing and types of monitoring activities are not specified by A.S.P.E.N., and there is no recognition in either guideline of the interdisciplinary team being involved in preventative practices that support food intake.

Of the algorithms or guidelines the INPAC has been compared to here, only two have gone through systematic development based on grading the current evidence [16, 17], but none have used rigorous methods to develop consensus beyond this evidence. The ESPEN screening guideline does not describe the process of its development [18]. The more recent feedM.E. algorithm [19] is based on research and literature, yet evidence for some steps in their process, such as diet counselling and fortification of the diet following a positive screen, were not presented; most of the literature in this review was focused on oral nutritional supplements or nutrition support for treatment. Furthermore, only selected experts were included in the process to develop these guidelines and frontline staff, who support the nutrition care of their patients on a daily basis, were not included. The A.S.P.E.N. pathway was guided by the Board of Directors and was systematically developed from the best available evidence at the time [16]. The resulting pathway is based only on published quality evidence and this likely explains its lack of detail on areas included in the INPAC (e.g. importance of monitoring food intake). Furthermore, details on the systematic review for this literature is described but the process for developing the pathway itself and how consensus was reached is less clear [16]. It does however indicate that external experts reviewed the guideline and the Board of Directors approved the final version. The Dietetic Association of Australia guideline went a further step of engaging 95 stakeholder dietitians post creation [17], not to develop consensus, but rather to support understanding and uptake of the final product. Subsequent focus groups were used to identify barriers to uptake [17] and the guideline was sent to other stakeholders including other health professions for input, although response was limited [17]. Thus, the INPAC is unique not only in its content, but in its rigorous development.

The INPAC has several unique features that are considered best practice, which resulted from its thorough development. A strength of this work is that it was not limited to a systematic review process to develop the pathway. Although systematic reviews are beneficial for summarizing a body of literature, when this literature is absent, they unfortunately do not provide guidance. For example, weighing of patients at admission and periodically throughout their hospital stay is a best practice to support nutrition care [38, 39], but extant literature on the utility and benefits of a weight measure in acute care are absent and thus this practice was not used as a decision point in prior guidelines. This practice is included in the INPAC as a monitoring device because it was identified and rated highly in the consensus based process. The modified Delphi approach allowed for the inclusion of ‘better evidence’ through inclusion of diverse disciplines with their own perspectives and expertise on the necessary and feasible nutrition care practices for medical and surgical patients that should be included in this pathway.

The potential to modify the traditional Delphi process is another advantage of this method for developing consensus; a variety of modifications especially around the first round for establishing the issue that requires consensus have been used [40, 41]. Further strengths of this methodology were: the use of a rigorous approach for developing the structured questions for the Delphi using the stakeholder meeting points of discussion; the anonymous input to develop consensus; and, having a research assistant (JM) monitor the Delphi and summarize results to ensure that bias was minimized [30]. Consensus was reached on almost all components of the INPAC, and where consensus was not reached, flexibility in the pathway resulted. The inclusion of diverse stakeholders across regions and high retention for both rounds of the Delphi are further strengths of this work [30]. Engagement by participants was also evident in the extent of qualitative comments to clarify ratings on items. Finally, a short time frame between rounds to maintain interest and commitment of participants helps to encourage completion [30]; we were able to achieve a short turnaround time due to dedicated personnel who summarized results and created standardized questions for the second round, resulting in minimal drop-out. In addition to the modified Delphi, further opportunities to face validate the pathway were undertaken. Focus groups and the final stakeholder meeting resulted in format changes to support interpretation by various clinical groups.

The key limitation with any consensus approach is the experts included and their opinions and biases [30]. There is no ideal size for participants in a Delphi survey [28, 30]. Smaller groups tend to be more homogenous, resulting in a potentially limited view of consensus, whereas larger groups often have a range in depth of expertise, resulting in the development of only general statements that achieve consensus [30]. Participants for this modified Delphi did vary in expertise from CMTF investigators to practitioners new to the concept of a care pathway focused on nutrition screening, assessment and care practices of medical and surgical patients. Yet, all had experience in identifying malnutrition and nutritionally caring for patients. The initial stakeholder meeting, as well as background evidence-informed document, provided a common knowledge base. The four focus groups provided opportunity for external review and vetting of the resource with a wider group of clinical disciplines and varied experience with nutrition care being represented. The final stakeholder meeting helped to confirm and finalize the pathway. Future work should confirm this pathway for other countries and jurisdictions with varying acute care systems. However, the background evidence-informed document was based on international research and practice, and it is anticipated that core components of the INPAC are transferable to other health care systems.

## Conclusions

Malnutrition in acute care is a longstanding problem that has been resistant to change, primarily due to inadequate identification of malnourished patients and follow through of treatment. Several efficacious treatments exist to improve nutritional status, but changing the culture of care to promote nutrition and food intake is needed. The Integrated Nutrition Pathway for acute Care is a rigorously developed care pathway that is believed to be feasible in practice. Further research is needed to determine resource and implementation requirements to support further knowledge translation and uptake into practice.

## Abbreviations

CMTF: Canadian Malnutrition Task Force
INPAC: Integrated nutrition pathway for acute care
ONS: Oral nutritional supplements
SGA: subjective global assessment
